# A Case of Buccal Chancre

**Published:** 1871-05

**Authors:** 


A Case of Buccal Chancre.—E. Burke Haywood, M.D., Raleigh, N. C. (*Transactions North Carolina Medical Society*) recently saw a young lady suffering from buccal chancre, caused by a kiss given her on the lips by her engaged lover, who was at that time suffering from secondary syphilis. The contagion was communicated by the secretion from a mucous patch on the upper lip of the lover. He was ignorant of the danger of contaminating his lady-love by this manifestation of his affection. He had been under treatment several weeks before for primary syphilis, and thought that he was cured. The young lady, when first seen, had a well-marked chancre on her upper lip, which had existed for more than ten days, and was thought by her to have been caused by a chapped lip. There was much induration of the upper lip, and the parotid and submaxillary glands were much enlarged and very painful. The induration continued for several weeks, and was followed by well-marked secondary symptoms.—*Medical Record.*

